# The Application of a Nanomaterial Optical Fiber Biosensor Assay for Identification of *Brucella* Nomenspecies

**DOI:** 10.3390/bios9020064

**Published:** 2019-05-21

**Authors:** Kelly McCutcheon, Aloka B. Bandara, Ziwei Zuo, James R. Heflin, Thomas J. Inzana

**Affiliations:** 1 Department of Physics, College of Science, Virginia Tech, Blacksburg, VA 24061, USA; kmccutch@vt.edu (K.M.); dzwei.dzwo@gmail.com (Z.Z.); rheflin@vt.edu (J.R.H.); 2 Department of Biomedical Sciences and Pathobiology, Virginia-Maryland College of Veterinary Medicine, Virginia Tech, Blacksburg, VA 24061, USA; abandara@vt.edu; 3 Long Island University, Brookville, NY 11548, USA

**Keywords:** *Brucella abortus*, *Brucella melitensis*, *Brucella suis*, optical fiber, biosensor, nucleotide probe, light transmission, diagnosis

## Abstract

Bacteria in the genus *Brucella* are the cause of brucellosis in humans and many domestic and wild animals. A rapid and culture-free detection assay to detect *Brucella* in clinical samples would be highly valuable. Nanomaterial optical fiber biosensors (NOFS) are capable of recognizing DNA hybridization events or other analyte interactions with high specificity and sensitivity. Therefore, a NOFS assay was developed to detect *Brucella* DNA from cultures and in tissue samples from infected mice. An ionic self-assembled multilayer (ISAM) film was coupled to a long-period grating optical fiber, and a nucleotide probe complementary to the *Brucella* IS*711* region and modified with biotin was bound to the ISAM by covalent conjugation. When the ISAM/probe duplex was exposed to lysate containing ≥100 killed cells of *Brucella*, or liver or spleen tissue extracts from *Brucella-*infected mice, substantial attenuation of light transmission occurred, whereas exposure of the complexed fiber to non-*Brucella* gram-negative bacteria or control tissue samples resulted in negligible attenuation of light transmission. Oligonucleotide probes specific for *B. abortus*, *B. melitensis*, and *B. suis* could also be used to detect and differentiate these three nomenspecies. In summary, the NOFS biosensor assay detected three nomenspecies of *Brucella* without the use of polymerase chain reaction within 30 min and could specifically detect low numbers of this bacterium in clinical samples.

## 1. Introduction

Brucellae are bacterial pathogens responsible for brucellosis of domestic and wild animals and are zoonotic pathogens for humans. *Brucella* spp. are small gram-negative, nonmotile, aerobic, and slow-growing coccobacilli. Despite the recognition of brucellae as a single genospecies based on DNA-DNA hybridization studies, they are systematically classified based on host specificity. The main terrestrial nomenspecies are *B. abortus* (cattle), *B. melitensis* (goats and sheep), *B. suis* (pigs), *B. canis* (dogs), *B. ovis* (sheep), and *B. neotomae* (woodrats) [1,2]. In addition, *Brucella* spp. can also be isolated from marine mammals [3,4]. Human infections are acquired by consuming unpasteurized milk and dairy products or by direct exposure to animals and their carcasses. Human brucellosis resulting from direct exposure is primarily a disease of farmers, shepherds, veterinarians, microbiologists, butchers, and slaughterhouse workers [5,6].

Wild animals play an important role in the epidemiology of *Brucella* infections. *Brucella* spp. remain enzootic in wild elk and bison in the Greater Yellowstone region that includes areas of Montana, Idaho, and Wyoming. As a result, these animals are a reservoir for *B. abortus* in the United States [7]. Transmission of *Brucella* spp. to susceptible cattle normally occurs by ingestion or oral contact with infected fetuses that have been aborted, fetal fluids and membranes, or uterine discharges [8]. Elk that congregate on feeding grounds from November through April overlap with the peak time period when *Brucella* is transmitted to other animals (February through June) [9]. Maichak et al. reported that as many as 12% of the elk attending feeding grounds come into contact with non-infectious elk fetuses placed on these sites [10]. Bison normally congregate in large numbers, which increases the likelihood they will come into contact with *Brucella-*infected fetuses and discharges. Such congregation increases the possibility that infected bison could transmit *Brucella* to cattle herds in the area [7]. As a result, farmers may unnecessarily kill elk or bison that wander out of the park and onto private farmlands.

Development of reliable and cost-effective diagnostic tests for use in elk and other wildlife is a high research priority. Reliable and portable diagnostic assays that can be carried out in the field by non-specialist personnel are urgently needed to minimize the spread of the disease among wildlife and its transmission to domestic animals and humans.

Biosensors combine biological molecules with a physicochemical transducer. Biological components incorporated into biosensors may include nucleic acids, enzymes, antibodies, etc., and the transducer may be optical, electrochemical, thermometric, or piezoelectric. Regardless, the detection of the target biological material results in a measurable signal. The advantages of optical fibers (light, inexpensive, and low interference) have established them as essential instruments of sensor technology [11]. Biosensors that consist of optical fibers transmit light based on total internal reflection through their transduction elements. The sensor produces a signal that can be analyzed and is in proportion to the concentration of the molecule that binds to the biological element on the sensor. Grating devices in the optical fiber induce a periodic variation in the refractive index of the optical fiber’s core. As a result, there is a significant drop in the amount of light transmitted through the fiber at a specific wavelength. The specific wavelength can be changed to account for temperature, pressure, or the type of binding event [12].

Layer-by-layer films, also known as ionic self-assembled multilayer (ISAM) films, are a novel type of materials that enable the user to modify the structure and thickness of the thin film at nanometer levels. The assembly of such films is simple and inexpensive [13,14]. As a result, optical fibers containing these nanoscale overlays substantially enhance, through direct light transmission, the detection of antigen binding to antibody or DNA hybridizing to complementary DNA. Furthermore, these sensors can be organized into a device that is rugged and portable [15,16]. For the detection of infectious agents, these fiber-optic biosensors can be used as rapid diagnostic or screening tests prior to culture, serology, or other means of diagnosis. A variety of fiber grating-based biosensor platforms have recently been developed [17,18,19,20]. For the work described here, a nanomaterial optical fiber biosensor (NOFS) assay was successfully used to detect *Brucella* DNA in culture lysates and in infected animal tissues.

## 2. Materials and Methods

### 2.1. Oligonucleotide Primers and Probes

The oligonucleotide probes and primers (Table 1) were designed manually based on the DNA sequences of the respective genes/regions in GenBank and were purchased from Integrated DNA Technologies, Collinsville, IL, USA. The IS*711* DNA region is present in all known nomenspecies of *Brucella*, but not in other bacteria. Therefore, the primers IS*711*-For and IS*711*-Rev and probes IS*711*-BIO and IS*711*-DIG from the IS*711* region (Table 1) were used for detection of all *Brucella* nomenspecies and to distinguish them from other bacterial species. In order to identify and differentiate the three major *Brucella *nomenspecies (*Brucella abortus*,* B. melitensis*, and *B. suis*), the oligonucleotide probes BruAb2_0168 (GenBank accession AE017224.1), Melitensis_0466 (GenBank accession AE008918.1), and Suis_TraJ (GenBank accession CP024421.1) (Table 1) were used. A search using the NCBI BLAST program confirmed the specificity of the DNA regions used for identifying and distinguishing the respective *Brucella* species.

### 2.2. Bacterial Strains and Culture Conditions Used

The *Brucella* nomenspecies and other bacterial species used as controls in this study are listed in Table 2. The bacteria were grown to mid-log phase in brain heart infusion (BHI) broth. Bacteria were harvested by centrifugation, washed, resuspended in phosphate buffered saline, pH 7.2 (PBS), and killed by boiling for 20 min (confirmed by viable plate count). Serial dilutions of killed cell suspensions were made in PBS, and genomic DNA was harvested using the DNAeasy Blood and Tissue kit (Qiagen, Valencia, CA, USA).

### 2.3. PCR

PCR, when used, was performed in 25 μL volumes and included 10 pmol each of the primers Primer-IS*711*-For and Primer-IS*711*-Rev (Table 1), 1 mM MgCl_2_, 200 μM dNTPS, 1X concentration of One*Taq* Standard Reaction Buffer (New England Biolabs, Ipswich, MA, USA), 1.25 units of One*Taq* DNA Polymerase (New England Biolabs), and template DNA. Template DNA included either 26 ng of genomic DNA or 1 µL of heat-killed, cell lysate from 1 × 10^5^ cells/mL to 3 × 10^10^ cells/mL. Reaction conditions were an initial denaturation temperature of 95 °C for 5 min, followed by 30 cycles of 95 °C/1 min, 57 °C/1 min, 72 °C/1 min, and a final extension at 72 °C/10 min.

### 2.4. Enzyme-Linked Immunosorbent Assay (ELISA)

An ELISA was designed using Magnalink Streptavidin Magnetic beads (Solulink Inc., San Diego, CA, USA). The protocol was as described by the manufacturer (Solulink, Inc.) with modification. Briefly, 60 pmol of the biotinylated probe (Probe-IS*711*-BIO) (Table 1) in 250 μL of nucleic acid binding and wash buffer (50 mM Tris-HCl, 150 mM NaCl, 0.05% Tween 20, pH 8.0) was incubated for 30 min at room temperature with the beads in 1.5 mL microcentrifuge tubes. The heat-denatured PCR products or genomic DNA were incubated with the beads coupled to the biotinylated probe in hybridization buffer (3× SSC, 0.05% Tween 20) for 2 h at 45 °C. A digoxigenin-labeled probe (Probe-IS*711*-DIG) (Table 1) in hybridization buffer was then incubated with the bead/probe/DNA triplex for 2 h at 45 °C. The DIG Detection Starter Kit from Roche (Sigma-Aldrich, St. Louis, MO, USA) was used to determine binding of the probe to the triplex complex. The ELISA was designed solely to confirm that the designed probe would hybridize with the *Brucella* genomic DNA, and not as a diagnostic assay itself. Therefore, quantitative data were not obtained.

### 2.5. Fabrication of the ISAM Film

**Figure 1:**
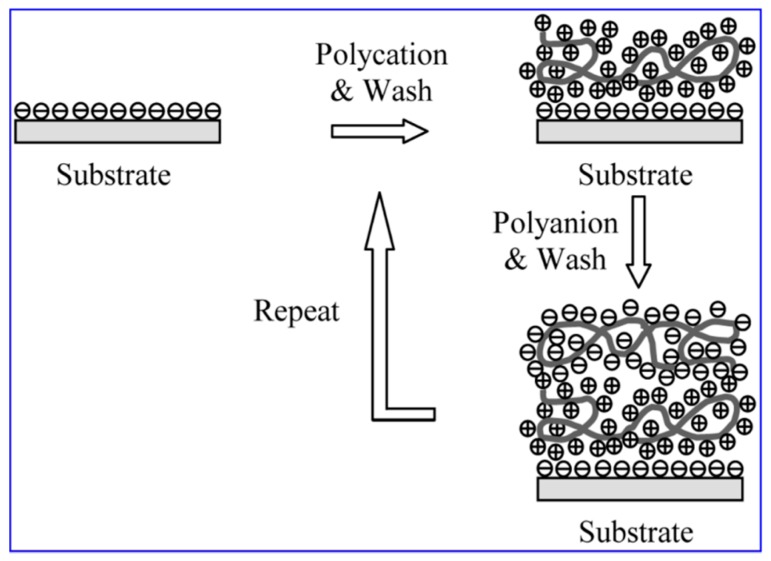
Assembly of the ionic self-assembled multilayer (ISAM) film. Polycationic and polyanionic solutions were alternately deposited on the optical fiber to form the ISAM film.

ISAM films were fabricated using procedures described by the authors [21]. The ISAM method simply involves the alternate dipping of a charged substrate (optical fiber) into an aqueous solution of a polycation and an aqueous solution of a polyanion at room temperature. The optical fiber was immersed in an aqueous 10 mM poly-allylamine hydrochloride (PAH) (pH 7.0) solution for 2 min then rinsed three times in distilled water. The fiber was then immersed in a similar aqueous solution of 10 mM poly-1-[p-(3′-carboxy-4′-hydroxyphenylazo) benzenesulfonamido]-1,2-ethanediyl (PCBS) (pH 7.0) for 2 min and rinsed again. The final layer was always the negatively-charged PCBS. These two steps were repeated until the optimal number of bilayers was obtained, which, for this assay, was four layers (Figure 1).

### 2.6. Coupling the Probe to the ISAM Film

The ISAM film was incubated with 0.6 mL of 0.17 M freshly prepared *N-*(3-dimethylaminodipropyl)-*N’-*ethylcarbodiimide (EDC), 0.17 M *N-*hydroxysulfosuccinimide (NHS), and 60 pmol of biotinylated oligonucleotide probe in PBS, pH 7.0, at room temperature for 30 min.

### 2.7. Conjugation of Streptavidin to the ISAM Film

An alternative method to couple the probe onto the ISAM film involved using a streptavidin intermediate. Four bilayers were deposited onto the optical fiber, leaving PCBS with negatively-charged carboxyl groups exposed. Then, 40 μL of streptavidin (1 mg/mL in PBS, pH 7.0) was mixed with 0.6 mL of cross-linker solution (0.17 M EDC and 0.17 M NHSS in PBS, pH 7.0). The mixture was added to the fiber and incubated for 8 h, with mixing every 15 min. The fiber was then rinsed and the biotinylated probe was added for spontaneous coupling to streptavidin.

### 2.8. NOFS Assay

The NOFS assay consists of turnaround point long-period gratings (TAP-LPGs) with ISAM films adsorbed on fiber cladding. The TAP-LPGs are TrueWave RS^TM^ (OFS) single-mode optical fibers with a grating period of 116 μm written by a 248 nm excimer laser through a chrome-plated mask. The grating couples to the LP_0,14_ cladding mode of the fiber. White light model SLD-11OESL003 (FiberLabs, Inc. Fugimino-Shi, Saitama, Japan) was coupled to the optical fiber, and the spectra were measured by an optical spectrum analyzer (ANDO AQ6317) following the deposition of materials onto the TAP-LPG.

**Figure 2:**
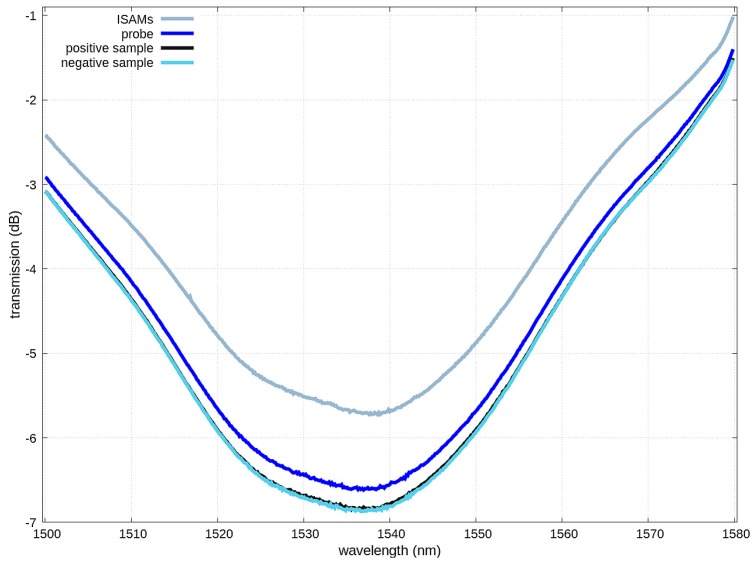
Transmission spectra after different steps of the assay. Adding the probe to the ISAM-coated fiber caused a large increase in attenuation. The attenuation was further increased after exposing the sensor to the positive control. However, further exposure of the fiber to the negative control did not result in any further change in attenuation, as was expected. As a result, the negative control spectrum overlaps and is indistinguishable from that of the positive control.

Bacteria grown in broth medium were harvested and washed in PBS. Serials dilutions of cultures were inoculated to agar medium to determine the colony forming units (CFU)/mL. *Brucella* cultures were lysed by boiling for 30 min. Loss of viability was confirmed by viable plate count before removing the bacteria from the biosafety level-3 laboratory. Prior to beginning the assay, preparations of genomic DNA, PCR products, and lysates of bacterial cells were boiled for 5 min. The film/probe duplex was incubated with the heat-denatured sample (genomic DNA, DNA regions amplified by PCR, amplified DNA from killed cells, dilutions of lysed cells, or dilutions of extracts of tissues from infected animals) for 50 min to allow hybridization between the probe and sample DNA to occur [21]. The TAP-LPG was tuned beyond the turnaround point such that the two narrow-band peaks merged into a single broadband peak that changed attenuation strength as the coupling between the core and cladding mode was modified by the addition of material to the cladding surface. As light in the range of 1400–1700 nm was transmitted through the ISAM fiber, an optical analyzer was used to record the attenuation in light transmission at 1550 nm. This attenuation in light transmission occurred due to the increase in coupling of light out of the core of the optical fiber due to sample DNA hybridizing to the DNA probe. An example of the series of spectra, as material binds to the cladding surface, is shown in Figure 2. The thickness of the ISAM films used is determined in order to set the attenuation at approximately half of the maximum attenuation that occurs before peak split into two separate narrowband peaks.

### 2.9. Detection of Brucella DNA in Tissues of Infected Mice

Groups of two female BALB/c mice each were inoculated intraperitoneally with 6 × 10^4^ CFU/mouse of *B. abortus *strain 2308,* B. melitensis *strain 16 M, or *B. suis *strain 1330. Two mice were injected with PBS as controls. One week after inoculation the mice were euthanized, and 0.1 g of spleen and liver samples were collected. The tissues were ground with 1 mL of PBS. Half of the volume of the extracts of the ground tissues were used in viable plate count determination to determine the number of bacteria/g of tissue. The remaining half (500 µL of heat-denatured cell-free extract corresponding to 0.05 g of tissue) was used per each run of the NOFS assay, as previously described [21]. DNA in these samples were not amplified by PCR prior to NOFS testing. Serial dilutions of the extract were also cultured onto BHI agar, and bacterial colony counts were determined as CFU after 72 h of incubation at 37 °C with 5% CO_2_.

### 2.10. Statistical Analyses

The standard deviations of the means were calculated from assays repeated at least three times. The online calculator (http://www.danielsoper.com/statcalc3/calc.aspx?id=43) was used to determine the analysis of variance, which was used to compare the transmission attenuation between different samples. The online calculator (http://www.socscistatistics.com/tests/studentttest/Default2.aspx) was used to calculate *p*-values from the Student *t-*test and to compare the attenuation of light transmission recorded for infected versus control tissue extracts. Student *t-*tests were also used for analysis of the attenuation of light transmission after exposure of the probe to two different PCR products. Results with calculated *p-*values of less than 0.05 was considered significant. The cutoff value in percent attenuation of light transmission that was used to differentiate negative from positive samples was calculated by multiplying the standard deviation of the true negative isolates tested by 3. This cutoff value could change depending on the optical fiber used and varied from 0.6% light attenuation to 3.2% light attenuation. Larger cutoff values were due to larger standard deviations of the negative controls.

## 3. Results

### 3.1. Specificity of the DNA Probes

**Figure 3:**
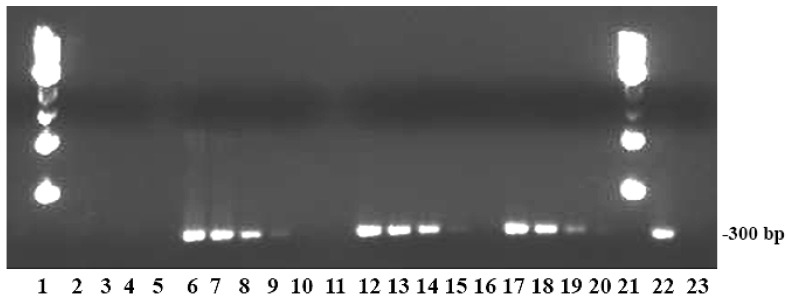
PCR amplicons from *Brucella* and control strains. Lanes and lysates representing the number of cells from nomenspecies used for PCR: 1 and 21, molecular size standards; 5 and 11, blank wells; 2–4, 3 × 10^7^, 3 × 10^5^, and 3 × 10^3^ cells of *Pseudomonas aeruginosa* (the same negative results for the same number of cells are not shown for *Escherichia coli* and *Salmonella* Typhimurium); 6–10, 8 × 10^6^, 8 × 10^5^, 8 × 10^4^, 8 × 10^3^, and 8 × 10^2^ cells of *B. abortus*; 12–16, 6 × 10^6^, 6 × 10^5^, 6 × 10^4^, 6 × 10^3^, and 6 × 10^2^ cells of *B. suis*; 17–20, 5 × 10^6^, 5 × 10^5^, 5 × 10^4^, and 5 × 10^3^ cells of *B. melitensis*; 22, positive control (26 ng of *B. abortus* genomic DNA; 23, negative control.

DNA amplification of *Brucella* and heterologous species using oligonucleotide primers to the IS*711* region (Table 1) confirmed the specificity of the IS*711* region for *Brucella* nomenspecies. An approximately 300 bp-size amplicon was obtained when 50 ng of genomic DNA or lysates containing at least 5 × 10^3^ killed cells of each *Brucella* nomenspecies was used in PCR reactions. However, visible amplicons were not seen in agarose gels when lysates representing 8 × 10^2^ or fewer *Brucella* cells were used in PCR reactions. PCR amplicons were also not seen when lysates containing up to 3 × 10^7^ killed cells of *Escherichia coli*, *Pseudomonas aeruginosa*, or *Salmonella* Typhimurium (negative controls) were used (Figure 3).

### 3.2. Validation of Target DNA for Hybridization to the DNA Probe

**Figure 4:**
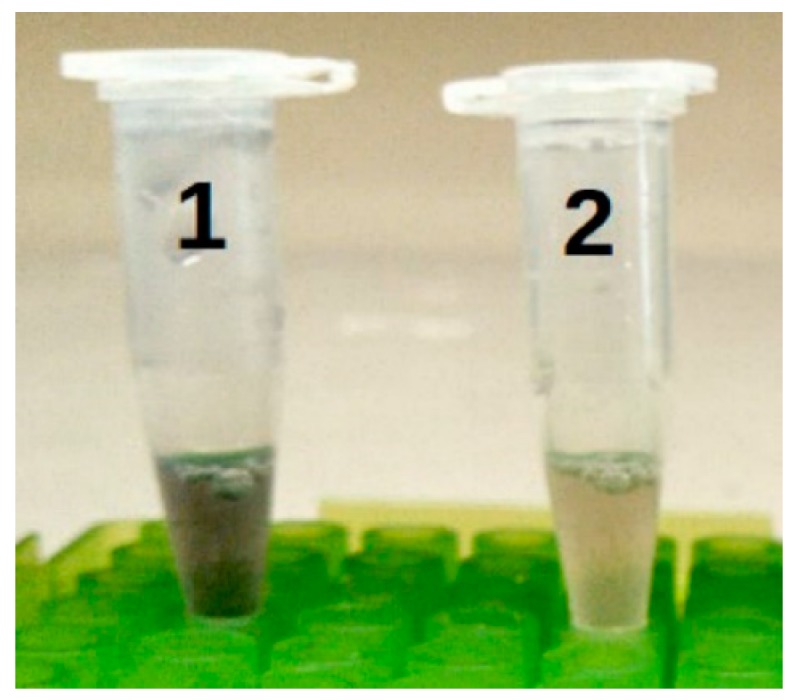
Magnetic bead ELISA. All tubes contained the beads, biotinylated probe of IS*711* gene, and digoxigenin-labelled probe to a distinct IS*711* DNA region. Tube 1 contained the PCR amplicon from a lysate of 7 × 10^6^ cells of *Brucella abortus*. Tube 2 contained all the reaction components of tube 1, but the genomic DNA was not amplified by PCR.

An ELISA was used to validate that target DNA hybridized to probes of the IS*711* region. After the DNA and initial bead-bound probe were allowed to hybridize, a second DIG-labeled oligonucleotide IS*711* probe to a different region of the DNA was added. Only if the sample DNA bound to the first probe would the second DIG-labelled probe bind and specifically detect *Brucella* DNA. The use of genomic DNA or lysate of killed cells in the absence of PCR did not produce a colorimetric change, indicating there was inadequate complementary DNA sequence from the genomic DNA to be detected in this assay. However, following DNA amplification of the test sample (genomic DNA or lysate containing 8 × 10^3^ cells of *Brucella*), a positive reaction was obtained (Figure 4), but not if lysate representing 8 × 10^2^ or fewer *Brucella* cells were tested (not shown). These results were consistent with results obtained by gel electrophoresis of PCR products and confirmed that the probe successfully bound to amplified DNA from the IS*711* region and was valid for use in the NOFS assay.

### 3.3. Identification of Brucella Nomenspecies by NOFS Assay

**Figure 5:**
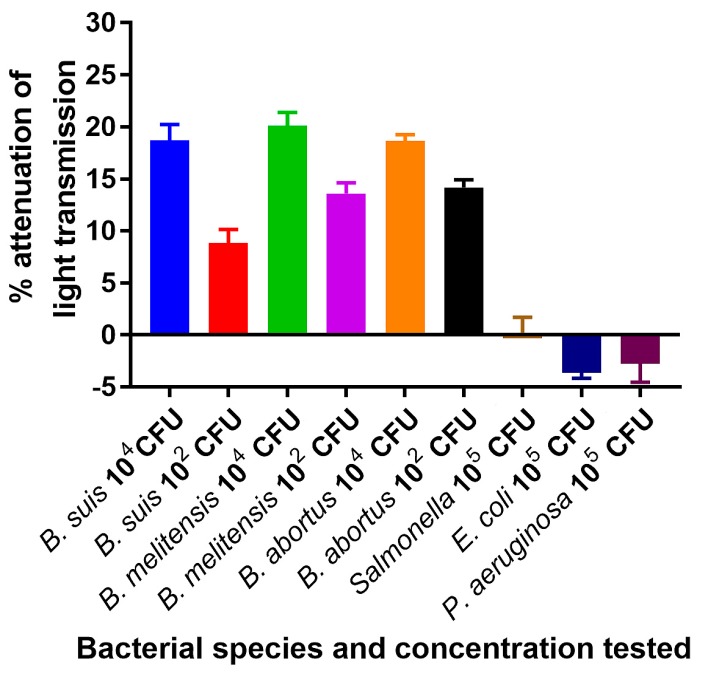
Detection of *Brucella* DNA amplified by PCR from *B. suis*, *B. abortus*, and *B. melitensis* by nanomaterial optical fiber biosensors (NOFS) assay. Each experiment consisted of 3 sequential steps: The biosensor was first tested with sample amplified by PCR from a lysate containing 10^4^ cells of a negative control strain (*E. coli*, *Salmonella*, or *P. aeruginosa*), followed by an amplified sample of lysate from 10^4^ *Brucella* cells, then lysate from an amplified *Brucella* culture containing 10^2^ cells.

Reaction of the ISAM/probe (IS*711*) duplex with the entire 25 µL of PCR amplicons from a lysate representing 10^4^ cells of *B. abortus *strain 2308, *B. melitensis* strain 16 M, or *B. suis *strain 1330 in 500 µL of PBS resulted in 18.7%, 18.6% and 20.11% attenuation of light transmission, respectively, with positive results being above 0.6% light attenuation. When lysate from 10^2^ cells of these same nomenspecies were tested, 8.8%, 14.2% and 13.6% attenuation of light transmission was obtained, respectively (Figure 5). These results indicated that the NOFS assay was capable of detecting PCR products with at least 10^2^ cells of *Brucella*, which is much lower than the number of cells that could be detected by gel electrophoresis or ELISA. In contrast, when lysate from 10^4^ cells of *P. aeruginosa*,* E. coli*, or* S.* Typhimurium were tested by the NOFS assay following PCR, less than 0.2% attenuation of transmission was obtained for any of the non-*Brucella* species tested (Figure 5).

**Figure 6:**
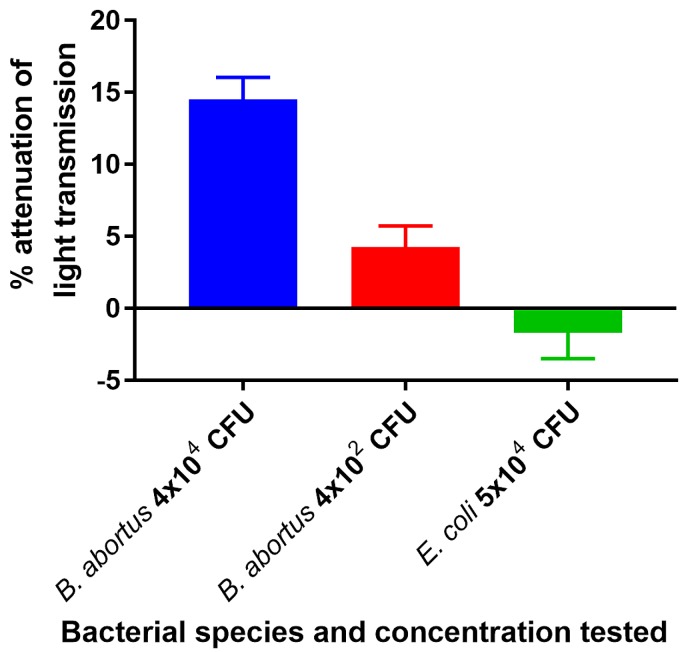
Detection of *B. abortus* DNA from lysates of killed cells by NOFS assay without PCR amplification. This experiment consisted of 3 sequential steps: The biosensor was first tested with lysate representing 5 × 10^4^ cells of a negative control strain (*E. coli*), followed by lysate containing 4 × 10^4^ cells of *B. abortus*, followed by lysate containing 4 × 10^2^ cells of *B. abortus*.

Reaction of the ISAM/IS*711* probe duplex containing streptavidin with lysate representing 4 × 10^2^ or 4 × 10^4^ cells of heat-killed *B. abortus* without the use of PCR resulted in 4.3% and 14.5% transmission attenuation, respectively. Reaction of the same duplex lysate representing 5 × 10^4^ cells of heat-killed *E. coli* failed to produce a positive transmission attenuation (Figure 6). These results confirmed that the assay could specifically detect low numbers of *Brucella* without the use of PCR.

When 10 replicates of each of the *Brucella* nomenspecies above were tested with lysates containing 10^2^ cells/reaction by NOFS with streptavidin and without PCR, all were positive for attenuation of light transmission and significantly greater in comparison to three different non-*Brucella* species tested in duplicate as negative controls (*p* ≤ 0.0004, pooled averages). The average light attenuation for *B. abortus* was 3.81% ± 0.92%, for *B. suis* was 3.50% ± 1.15%, and for *B. melitensis* was 5.15% ± 1.63% (all above the respective cutoff value for a positive result). Light attenuation results using lysates containing 10^4^ cells/reaction of the negative control species *E. coli*, *P. aeruginosa,* and *Salmonella* were 0.41% ± 1.28%, 0.93% ± 1.68%, and 1.04% ± 0.89%. These results could be obtained in 30 min and confirmed that the NOFS assay was a highly sensitive, specific, and rapid assay for the detection of *Brucella* DNA.

### 3.4. NOFS Assay to Detect and Distinguish Different Brucella Nomenspecies and to Distingusih Brucella from Non-Brucella Bacterial Types

When the ISAM/probe BruAb2_0168 complex (specific for *B. abortus* but not for other *Brucella* nomenspecies) was reacted directly with lysate containing 10^5^ heat-killed cells of *B. abortus* strain 2308 (without PCR), light transmission was attenuated by 5.4%. However, when the same ISAM/probe complex was reacted with a similar number of *B. melitensis* 16 M cells, transmission was attenuated only by 0.2%. In a separate assay, when the ISAM/probe Suis_*TraJ* complex (specific for *B. suis*) was reacted with lysate containing 10^5^ cells of *B. suis *strain 1330, light transmission was attenuated by 3.8%. However, when the same ISAM/probe complex was reacted with lysate containing 10^5^ cells of *B. abortus*, no positive attenuation of transmission was observed. When the ISAM/probe IS*711* complex was reacted with lysate representing 10^5^ cells of 15 non-*Brucella* bacterial samples (Table 3), less than 2.2% light transmission attenuation was observed (all below the respective positive cutoff value). Thus, the NOFS assay was specific for *Brucella* and could detect and distinguish different *Brucella* nomenspecies.

### 3.5. Identification of Brucella in Tissues from Infected Mice by NOFS Assay

When the ISAM/probe BruAb2_0168 complex (specific for *B. abortus*) was reacted with 2 spleen or 2 liver extracts from *B. abortus-*infected mice, light transmission was attenuated by 6.79% ± 0.34% and 3.38% ± 0.78%, respectively (positive values were above 3.2% light attenuation for these assays). The average bacterial loads in the spleen and liver extracts used in the assays were 3.8 × 10^4^ and 4 × 10^3^ cells, respectively. However, when the same ISAM/probe complex was reacted with 2 spleen or 2 liver extracts from control mice inoculated with PBS, there was no positive attenuation of transmission for any of the samples (mean attenuation was −1.47% and −1.78%, respectively). Therefore, the NOFS assay could detect *B. abortus* in infected mouse spleen and liver. When the ISAM/probe Melitensis_0466 (specific for *B. melitensis*) was reacted with 2 spleen or 2 liver extracts from *B. melitensis-*infected mice, light transmission was attenuated by 7.6% and 6.1%, respectively. However, when the same ISAM/probe complex was reacted with 2 spleen or 2 liver extracts from PBS-injected mice, positive attenuation of transmission was not seen. Similarly, when the ISAM/probe Suis_TraJ complex (specific for *B. suis*) was reacted with 2 spleen or 2 liver extracts from *B. suis-*infected mice, light transmission was attenuated by 6.9% and 5.1%, respectively, but light transmission was less than 1% when the probe complex was reacted with 2 spleen or 2 liver extracts from PBS-injected mice (Table 4).

## 4. Discussion

Biosensors are becoming established diagnostic modalities and, when combined with PCR, have been used for detection of DNA using impedance spectroscopy [22,23] and piezoelectric gold electrode [24]. However, such biosensors are very expensive (i.e., may cost over $10,000 apiece), require high maintenance by experienced personnel, and have the additional PCR step. Therefore, these assays may also not be practical for many laboratories. Optical transduction methods such as surface plasmon resonance (SPR) are rapid and sensitive devices that have been developed for detection of bacterial agents [25]. However, these assays require the use of LED and spectroscopy to generate excited light and receive a signal. SPR sensors are also expensive and require highly trained personnel. Unlike other published biosensors, the NOFS assay described here utilizes nanometer-thick layers that can include a variety of materials, such as DNA, antibodies, and antigens. TAP-LPGs that are coupled to a DNA probe specific to the bacterium and supplemented with additional layers of biotin-streptavidin further enhance the limit of detection of the assay. As a result, PCR was not required for adequate detection of DNA with this NOFS assay. When the target DNA binds to the complementary oligonucleotide probe, thus altering the thickness of the film, the refractive index is also changed. As a result, the transmission characteristics of the fiber are modified, resulting in attenuation of the percent light transmitted. Due to the high specificity of the compatible DNA probe and target, specific DNA can be detected.

DNA probe assays have previously been used to identify the common nomenspecies of *Brucella* [26]. Specific primers have been used with DNA amplification to successfully differentiate *B. abortus* biovars 1, 2, and 4, *B. melitensis*, *B. suis* biovar 1, and *B. ovis*. [27,28]. Several investigators have shown that by targeting highly conserved genes (i.e., 16S rRNA [29], 16S-23S intergenic spacer regions [30], *bcsp*31 or IS*711* for all *Brucella* species [31,32], *alk*B for *B. abortus*, and BMEI1162 for *B. melitensis* [27,33]), probes and primers can be developed for direct detection of these agents. In this communication, DNA amplification was used to confirm that the IS*711* DNA region was specific for all three nomenspecies of *Brucella*, and an ELISA was used to demonstrate that the oligonucleotide probe specifically binds to the IS*711* region of *Brucella.* The NOFS assays, which included a biotin-streptavidin linker, were used to detect as few as 100 cells of *Brucella* with a high degree of sensitivity and specificity in the set of samples studied here, even without prior PCR amplification. The NOFS assay was also capable of detecting *Brucella* in the tissues of infected mice. The probes to BruAb2_0168, Melitensis_0466, and Suis_TraJ DNA regions were specific for *B. abortus*, *B. melitensis*, and *B. suis*, respectively, as determined by NOFS. Therefore, these designed oligonucleotide probes could be used to distinguish each of the respective *Brucella* nomenspecies from each other or heterologous bacteria. Major advantages of the NOFS assay were that it could be completed in less than 1 h, did not require particular expertise to perform, and did not require a large amount of bench space.

The detection of antibodies to the lipopolysaccharide (LPS) O-antigen by serological methods is the accepted diagnostic method for brucellosis in all hosts. However, specificity can be problematic due to the structural similarity of the O-antigen side chain of *Brucella* with that of other bacteria, particularly *Yersinia enterocolitica* O:9, *Vibrio cholerae*, and *E. coli* 0:157. At this time, no other antigens have been identified that can successfully replace the LPS O-antigen in diagnostic assays. Molecular diagnostic tests are now important methods in clinical microbiology, although they remain restricted to larger laboratories that have the funds, expertise, and equipment to utilize this technology. Real-time (q)PCR assays can detect the DNA of infectious disease agents with same day results [34,35,36]. However, qPCR technology is restrictive due to the large cost of equipment and expertise needed to carry out these assays. Therefore, qPCR is normally not available in medical settings that have or utilize small laboratories, particularly in rural communities where infections due to *Brucella* may be more prevalent. Furthermore, *Brucella* can be exceptionally difficult to detect in blood, and although isolation from animal tissues may be more productive, as we show here by detection of *Brucella* by NOFS from mouse tissues, such isolation is normally not practical with human tissues. Nonetheless, the NOFS assay can be modified to detect antibodies to *Brucella* by coupling the antigen to the fiber, rather than a DNA probe, or alternatively coupling antibodies to the fiber to detect a specific antigen. We have described such an assay using antibody-coupled sensors to detect methicillin-resistant *Staphylococcus aureus* [21] and *Francisella tularensis* [37].

The most prominent reservoir of *B. abortus* in the United States in in bison and elk in the Yellowstone National Park area [7]. Cattle farmers are particularly concerned that bison or elk that wander onto their farmlands may be infected with *Brucella* and transmit the agent to their cattle, resulting in their loss of *Brucella*-free status. The NOFS assay has the advantage that samples collected from anesthetized animals or aborted fetuses could be used in a small regional facility to rapidly detect the presence of *B. abortus*. In addition, *B. suis* is the most prevalent *Brucella* nomenspecies in the United States and is present in feral hogs in 14 U.S. states [38]. *B. suis* can be transmitted to humans through hunting (field dressing and butchering) or other close contact [39]. Although *Brucella* diagnosis in humans can be difficult due to non-specific flu-like symptoms, detection of the agent in the animal’s tissues can strongly support the diagnosis. Therefore, with the correct primers and probes, this NOFS assay can be adapted to detect any of the *Brucella* nomenspecies.

The NOFS assay described here is at the proof-of-principle stage. Further work will be required to develop a diagnostic test ready for regulatory approval. Such work will require a large number of *Brucella* strains of the different nomenspecies, as well as additional negative control strains, to adequately determine the sensitivity and specificity of the assay. Although the limit of detection of the assay has been determined (about 100 cells/mL), due to the low number of strains of *Brucella* available to us, sensitivity (defined as the true positive rate divided by false positives) was not able to be accurately calculated. Although the specificity of the assay appeared to be 100%, additional control strains encompassing a wide variety of species would need to be tested to confirm this. In addition, the NOFS assay can easily be modified to detect antigens, antibodies, or DNA from a wide variety of infectious agents, including viruses, fungi, and parasites, as well as other bacteria. In addition, the assay could be used to detect DNA encoding for antibiotic resistance genes to aid in screening patients that may be colonized with bacteria carrying specific antibiotic resistance genes. Such an assay would be highly beneficial in hospital infection control situations, particularly with an assay that can be completed in a short period of time for reasonable cost, and without the need for highly trained personnel.

## Figures and Tables

**Table 1:** Oligonucleotide probes and primers used for detecting major *Brucella* nomenspecies.

Probe/Primer Name	Sequence (5′ to 3′)	Comments
Probe-IS*711*-BIO	AAGCCAACACCCGGCCATTATGGT	The probe was biotinylated at the 3′ end
Probe-IS*711*-DIG	GGCCTACCGCTGCGAATA	The probe was labeled with digoxigenin at the 5′ end
Primer-IS*711*-For	TTGGCCTTGATCTGAGCCGT	
Primer-IS*711*-Rev	ATCGAAAGTCCACGCAGATG	
Probe-BruAb2_0168	TGGAACGACCTTTGCAGGCGAGATC	Biotin label at the 3′ end; specific for *B. abortus*
Probe_Melitensis_0466	CCAGCTTTTGGCCTTTTCCAGATTG	Biotin label at the 3′ end; specific for *B. melitensis*
Probe_Suis-TraJ	CCATGAGCGCCCGCATGTCCTCTTG	Biotin label at the 3′ end; specific for *B. suis*

**Table 2:** Bacterial species and strains used.

Bacterial Species	Strain Name	Source
*Brucella abortus*	2308	Virginia Tech Biosafety 3 laboratory
*Brucella melitensis*	16 M	Virginia Tech Biosafety 3 laboratory
*Brucella suis*	1330	Virginia Tech Biosafety 3 laboratory
*Klebsiella pneumoniae*	2237	Virginia Tech Veterinary Teaching Hospital
*Klebsiella pneumoniae*	2237-13	Virginia Tech Veterinary Teaching Hospital
*Proteus mirabilis*	2172B	Virginia Tech Veterinary Teaching Hospital
*Proteus mirabilis*	13-2319	Virginia Tech Veterinary Teaching Hospital
*Proteus mirabilis*	13-2401	Virginia Tech Veterinary Teaching Hospital
*Staphylococcus aures*	29213	Virginia Tech Veterinary Teaching Hospital
*Enterococcus faecalis*	2174	Virginia Tech Veterinary Teaching Hospital
*Enterococcus faecalis*	13-2321-2	Virginia Tech Veterinary Teaching Hospital
*Escherichia coli*	2174	Virginia Tech Veterinary Teaching Hospital
*Escherichia coli*	Top10	ThermoFisher Scientific
*Escherichia coli*	13-2438	Virginia Tech Veterinary Teaching Hospital
*Salmonella arizonae*	13-2453	Virginia Tech Veterinary Teaching Hospital
*Enterobacter aerogenes*	13-2329-2	Virginia Tech Veterinary Teaching Hospital
*Enterococcus faecium*	13-2174-C	Virginia Tech Veterinary Teaching Hospital
*Enterococcus faecium*	13-2248-2	Virginia Tech Veterinary Teaching Hospital

**Table 3:** Percent NOFS light attenuation from non-*Brucella* bacterial types.

Bacterial Species	Strain Name	Transmission Attenuation (Mean ± Standard Deviation)
*Klebsiella pneumoniae*	2237	−1.67% ± 0.15%
*Klebsiella pneumoniae*	2237-13	2.09% ± 2.63%
*Proteus mirabilis*	2172B	0.65% ± 0.31%
*Proteus mirabilis*	13-2319	0.70% ± 1.64%
*Proteus mirabilis*	13-2401	0.25% ± 0.95%
*Staphylococcus aures*	29213	1.30% ± 0.70%
*Enterococcus faecalis*	2174	1.57% ± 1.03%
*Enterococcus faecalis*	13-2321-2	1.04% ± 0.85%
*Escherichia coli*	2174	0.23% ± 0.06%
*Escherichia coli*	Top10	−0.62% ± 0.94%
*Escherichia coli*	13-2438	1.54% ± 0.12%
*Salmonella arizonae*	13-2453	−0.59% ± 1.27%
*Enterobacter aerogenes*	13-2329-2	1.64% ± 0.46%
*Enterococcus faecium*	13-2174-C	2.14% ± 0.10%
*Enterococcus faecium*	13-2248-2	1.69% ± 0.38%

**Table 4:** Percent NOFS ^a^ light attenuation from spleen and liver extracts from mice infected with *Brucella* spp.

Challenge	Organ	Average % Attenuation ± Standard Deviation	Test Result ^b^
With probe BruAb2_0168 (specific for *B. abortus*) *B. abortus*	Spleen (from mice 1 and 2)	6.80 ± 0.32	Positive
	Liver (from mice 1 and 2)	3.38 ± 0.78	Positive
PBS	Spleen (from mice 7 and 8)	−0.74 ± 1.12	Negative
	Liver (from mice 7 and 8)	−1.78 ± 0.29	Negative
With probe Melitensis_0466 (specific for *B. melitensis*) *B. melitensis*	Spleen (from mice 3 and 4)	7.60 ± 1.86	Positive
	Liver (from mice 3 and 4)	6.13 ± 2.81	Positive
PBS	Spleen (from mice 7 and 8)	−1.78 ± 2.60	Negative
	Liver (from mice 7 and 8)	−0.64 ± 0.41	Negative
With probe Suis_TraJ (specific for *B. suis*) *B. suis*	Spleen (from mice 5 and 6)	6.94 ± 0.59	Positive
	Liver (from mice 5 and 6)	5.10 ± 1.45	Positive
PBS	Spleen (from mice 7 and 8)	0.72 ± 2.93	Negative
	Liver (from mice 7 and 8)	0.34 ± 1.59	Negative

^a^ All samples included streptavidin as a linker in the NOFS assay. ^b^ The positive cutoff value for this assay was 3.2%.
